# Editorial: Lipids in the Brain

**DOI:** 10.3389/fneur.2020.00712

**Published:** 2020-07-17

**Authors:** Elisabetta Albi, Alice Vladimirovna Alessenko, Fanny M. Elahi, Maria Dolores Ledesma

**Affiliations:** ^1^Department of Pharmaceutical Sciences, University of Perugia, Perugia, Italy; ^2^Laboratory of Chemical Physics and Bioanalytical Processes, Emanuel Institute of Biochemical Physics, Russian Academy of Sciences, Moscow, Russia; ^3^Department of Neurology, Memory and Aging Center, Weill Institute for Neurosciences, University of California, San Francisco, San Francisco, CA, United States; ^4^Severo Ochoa Molecular Biology Center (CSIC-UAM), Madrid, Spain

**Keywords:** Alzheimer, lipid, sphingolipid, cholesterol, neurodegeneration

Until about 30 years ago, lipids were thought to only play a structural role in cell membranes. Over time they have acquired a new face and today represent a new frontier research aimed at studying them as biologically active molecules essential for cell fate, especially in the central nervous system (CNS).

This special issue includes nine papers, five reviews, and four research articles, covering some of the more recent and exciting findings about the role in the brain physiopathology.

The first paper of the special issue is a review written by Corraliza-Gomez et al. “Lipid-Binding Proteins in Brain Health and Disease” that summarizes a systematic literature search to identify lipid interacting proteins and the biological processes in which they are involved related to nervous system function and dysfunction. Lipid-binding scaffolding proteins, membrane transporters and receptors, and transcription regulators contribute to blood brain barrier crossing and maintenance, control of oxidative stress and inflammation, organelle function, vesicle trafficking, myelin management, and amyloid dynamics. When altered, they may become therapeutic options.

The minireview by Torres et al. “Mitochondrial Cholesterol in Alzheimer's Disease and Niemann–Pick Type C Disease” illustrates the case of the aberrant accumulation of cholesterol in mitochondria as a common pathological event in Alzheimer disease (AD) and Niemann Pick type C. Mitochondrial cholesterol accumulation alters membrane physical properties and impairs the transport of glutathione into this organelle disrupting the antioxidant defense. The potential role of the up- regulation of a family of lipid transporting proteins containing StAR-related lipid transfer domains (StARD), by different mechanisms in the two diseases, is discussed.

The minireview of Isacson et al. “Novel Results and Concepts Emerging From Lipid Cell Biology Relevant to Degenerative Brain Aging and Disease” describes biochemical and clinical evidence of an interdependence of lipids and proteinpathy in neurodegenerative diseases (NDD). The authors illustrate that Parkinson's disease (PD) can be triggered by lipid disturbances caused by lysosomal genetic or similar age-induced enzymatic loss of function. The lipid-carrying apolipoprotein E4 variant is associated with increased risk for dementias and α-synuclein may even have a cooperative role with apolipoproteins and lipid transport.

Given the biological links between sphingolipids and brain injury, Azizkhanian et al. in “Plasma Lipid Profiling Identifies Biomarkers of Cerebral Microvascular Disease” investigate the biomarker value of plasma sphingolipids as a non-invasive marker of chronic subclinical cerebral small vessel disease. To this end they quantify the association of plasma sphingolipid (SphLs) levels with white matter hyperintensity volumes on brain magnetic resonance imaging, the current imaging gold-standard for cerebral small vessel disease. They find that levels of certain plasma SphLs are highly associated with white matter hyperintensities.

As the relevance of lipid alterations is confirmed for the pathology of numerous brain diseases the use of lipid levels as diagnostic tool gains interest. Wai Kin Wong et al., in “Comparison of Single Phase and Biphasic Extraction Protocols for Lipidomic Studies Using Human Plasma” compare the efficacy of different lipid extraction protocols for lipidomic studies. Using human plasma from normolipidomic adult volunteers they show that the recently developed single phase Alshehry method, which also has the advantage of avoiding the use of chloroform, is more effective than established biphasic methods particularly in the extraction of polar lipids.

The following paper spans the association between lipid dysmetabolism with complex psychiatric symptomatology. Taking an innovative approach to the study of acid sphingomyelinase pathways in comorbid psychiatric symptoms of depression-alcoholism, Kalinichenko et al. in “Enhanced Alcohol Preference and Anxiolytic Alcohol Effects in Niemann-Pick Disease Model in Mice” use acid sphingomyelin-knockout mice to show that forced alcohol consumption decreases anxiety while increasing depression in these models.

Martin-Segura et al. in “Aging Increases Hippocampal DUSP2 by a Membrane Cholesterol Loss-Mediated RTK/p38MAPK Activation Mechanism” study a mechanism that would link age-associated cholesterol changes to the chronic sterile inflammation when homeostatic control of kinases is lost. The authors provide compelling findings on the involvement of p38MAPK activity and its downstream targets in the hippocampus of old mice. The work presented builds on this to show that age-dependent loss of membrane cholesterol takes part in a negative-feedback loop that keeps p38MAPK activity levels within physiological range.

The next review “Yin-Yang Mechanisms Regulating Lipid Peroxidation of Docosahexaenoic Acid and Arachidonic Acid in the Central Nervous System” by Yang et al. provide information on the role of Yin-Yang mechanism in the release of arachidonic acid and docosahexaenoic acid due to different phospholipases A_2_ and in the regulation on their lipid peroxidation. The products of lipid peroxidation may be detected in body organs and fluids and may confer cytotoxic or protective effects depending on the conditions for production. The review reports in detail the involvement of these lipids in neurological and inflammatory pain, cerebral ischemia and brain injury, several neurodegenerative and neuropsychiatric disorders.

The review “Exploring Sphingolipid Implications in Neurodegeneration” by Alessenko and Albi provides evidence of participation of SphLs in the pathogenesis of AD, Parkinson's disease (PD) and, Amyotrophic Lateral Sclerosis (ALS). Recent studies have shown that SphLs play a decisive role in the neuronal function due to regulation of cell growth, differentiation, and death in the CNS. The review discusses results obtained *in vitro* and *in vivo*, such as brain tissue from both animals in which diseases were induced and humans in autopsy samples, liquor, and blood. It was highlighted that SphL species might be diagnostic markers and/or new targets for innovative therapeutic strategies.

Thus, the special issue gives an overview about the current knowledge and highlight interesting new insights into the roles of the complex world of lipids in the brain and on its involvement in NDD.

## Author Contributions

All authors listed have made a substantial, direct and intellectual contribution to the work, and approved it for publication.

## Conflict of Interest

The authors declare that the research was conducted in the absence of any commercial or financial relationships that could be construed as a potential conflict of interest.

